# A Simulated Graphical Interface for Integrating Patient-Generated Health Data From Smartwatches With Electronic Health Records: Usability Study

**DOI:** 10.2196/19769

**Published:** 2020-10-30

**Authors:** Jordan M Alpert, Naga S Prabhakar Kota, Sanjay Ranka, Tonatiuh V Mendoza, Laurence M Solberg, Parisa Rashidi, Todd M Manini

**Affiliations:** 1 Department of Advertising College of Journalism and Communications University of Florida Gainesville, FL United States; 2 Department of Computer & Information Science & Engineering University of Florida Gainesville, FL United States; 3 Department of Health Outcomes and Biomedical Informatics University of Florida Gainesville, FL United States; 4 College of Nursing University of Florida Gainesville, FL United States; 5 Geriatrics Research, Education and Clinical Center (GRECC) Veterans Health Administration, NF/SG VHS Gainesville, FL United States; 6 J Crayton Pruitt Family Department of Biomedical Engineering University of Florida Gainesville, FL United States; 7 Department of Aging and Geriatric Research University of Florida Gainesville, FL United States

**Keywords:** wearable, smartwatch, mobile, mHealth, user-centered design, electronic health records

## Abstract

**Background:**

Wearable technology, such as smartwatches, can capture valuable patient-generated data and help inform patient care. Electronic health records provide logical and practical platforms for including such data, but it is necessary to evaluate the way the data are presented and visualized.

**Objective:**

The aim of this study is to evaluate a graphical interface that displays patients’ health data from smartwatches, mimicking the integration within the environment of electronic health records.

**Methods:**

A total of 12 health care professionals evaluated a simulated interface using a usability scale questionnaire, testing the clarity of the interface, colors, usefulness of information, navigation, and readability of text.

**Results:**

The interface was positively received, with 14 out of the 16 questions generating a score of 5 or greater among at least 75% of participants (9/12). On an 8-point Likert scale, the highest rated features of the interface were quick turnaround times (mean score 7.1), readability of the text (mean score 6.8), and use of terminology/abbreviations (mean score 6.75).

**Conclusions:**

Collaborating with health care professionals to develop and refine a graphical interface for visualizing patients’ health data from smartwatches revealed that the key elements of the interface were acceptable. The implementation of such data from smartwatches and other mobile devices within electronic health records should consider the opinions of key stakeholders as the development of this platform progresses.

## Introduction

Wearable mobile technology enables long-term monitoring and capture of critical information about patients. Specifically, devices can be used to track physical activity, symptoms (eg, pain), and community mobility [1,2]. Health care professionals realize the value of receiving such data and have expressed the desire for those to be incorporated into electronic health record (EHR) systems [3]. However, simply adding data from wearable technology into EHRs can be problematic. Health care professionals were initially dissatisfied with the usability of EHRs when those systems were introduced [4,5], which led to difficulties in gaining proficiencies in EHR use [6] and slow adoption of the technology [7]. The best practices of implementation science indicate that involving stakeholders in the preimplementation and implementation phases to get their “buy-in” is necessary for success [8]. Involvement of stakeholders helps identifying user goals, which contributes to the acceptance and use of a system [9]. This study aims to test the usability of a graphical interface that displays patients’ health data from wearable devices (smartwatches) intended to be integrated within the EHR system by surveying health care professionals.

## Methods

### Setting and Study Design

**Figure 1:**
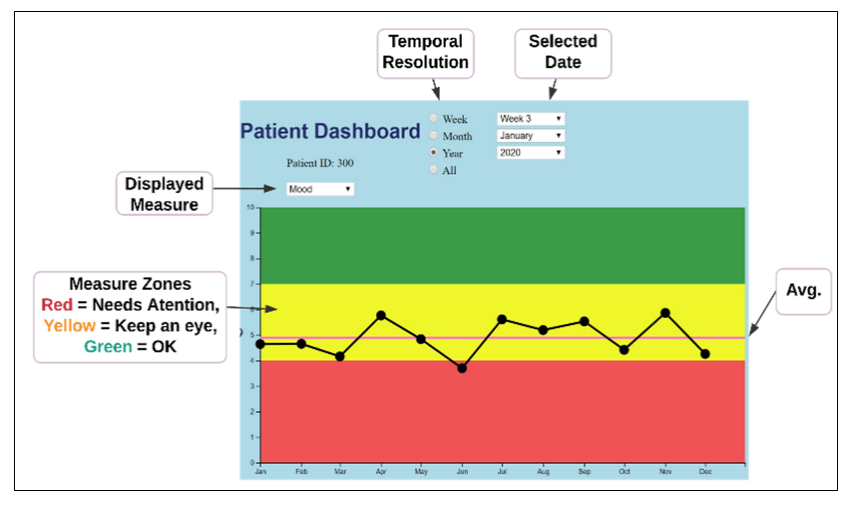
Simulated EHR dashboard. Avg: average.

Previously, a qualitative study was conducted with health care professionals about their perceptions and visual display preferences toward patient-generated data from smartwatches [3]. Based on the findings, a graphical EHR interface was developed to view measurements of attributes, such as pain, falls, hydration, and mobility patterns—the factors ranked high by health care providers in our previous study [3]. As part of the qualitative study, participants were aware that they would be recontacted to participate in the second phase. It is common for usability studies to repeat participants, as comparisons can be made to evaluate the efficacy of development [10-13]. All 12 participants from the qualitative study were recontacted via email to participate in this study, which focused on the usability of the interface. A link to an online survey with the sample interface was provided. First, participants were asked about the type of interface that would best suit their needs. Several figures were viewed, such as pie charts, bar graphs, and gauges; however, line graphs were most preferred due to their ability to display longitudinal data. Second, based on this information, a user interface was built using a web-based approach that would be suitable for an EHR interface (Figure 1). The interface mimicked what providers would see upon logging into an EHR system and allowed them to select the timeframe and specific variable. It was created on a separate server and was fully functional, which allowed users to toggle mock data as those would be received or summarized from smartwatches. The participants were queried again through an email that included 2 links. The first link directed participants to a simulated EHR interface with smartwatch data, and the second link led to the survey questionnaire (described in the next section). The survey instructions asked participants to respond to the questions after viewing and interacting with the simulated EHR interface for integration of health data from smartwatches.

### Usability and Data Analysis

The practice of usability testing is common with the presentation of graphical interfaces, and testing can enhance the efficiency of integrating EHR designs with existing workflow processes [14]. Thus, we evaluated the usability of the interactive elements and complex data presentation using a questionnaire developed by the International Organization for Standardization (ISO) to evaluate human-computer interactions (ISO 9241/110-S) [15,16]. This questionnaire contained 18 items. However, 2 items related to the ability to undo steps were not relevant to this interface and therefore were not evaluated. The remaining 16 items comprised 6 categories with the following principles: (1) suitability for the task, (2) conformity with user expectations, (3) self-descriptiveness, (4) controllability, (5) suitability for learning, and (6) error tolerance (Multimedia Appendix 1). Items focused on a variety of areas, including the clarity of the interface, colors, usefulness of information, navigation, and readability of text. An 8-point Likert scale ranging from 1 to 8 was used to gauge negative and positive sentiments toward each aspect of the interface. A score of 4 was considered neutral, consistent with another usability study that employed the same measurements as those used in this study [17]. The average scale scores and medians are presented in the next section along with the percent of responses above 5—the first green color code indicator, representing a positive score (as shown in Multimedia Appendix 1). In addition to evaluating individual categories, the ISO 9241/110-S evaluations also utilize aggregate scores, which range from 21 to 147 points [18].

## Results

### Participant Characteristics

There were 12 participants, representing different specialties, namely, geriatrics, orthopedic surgery, anesthesiology, nursing, and physical medicine and rehabilitation. The majority of participants were male (7/12, 58%) with an average age of 45 (SD 9.8) years. Health care professionals averaged 12 (SD 9.4) years of practice experience after residency. A detailed demographic summary is shown in Table 1.

**Table 1:** Demographic summary.

Characteristics		Values
**Sex,** **n (%)**		
	Female	5 (42)
	Male	7 (58)
**Age (years)**		
	Mean	45.1
	Range	33-64
**Years in practice**		
	Mean	12.4
	Range	4-35
**Race,** **n (%)**		
	White	8 (67)
	Indian	2 (17)
	Latino	1 (8)
	Asian	1 (8)
**Specialty,** **n (%)**		
	Geriatric	4 (33)
	Orthopedic surgery	4 (33)
	Anesthesiology	2 (17)
	Nursing	1 (8)
	Physical medicine and rehabilitation	1 (8)
**Patient setting,** **n (%)**		
	Outpatient	5 (42)
	Inpatient	3 (25)
	Both	4 (33)

### Evaluation Outcomes

Scores from 1 to 3 were interpreted as negative; score of 4 was considered neutral or average; and scores from 5 to 8 were considered as positive responses to the interface elements. Overall, the interface was positively received, with 14 out of the 16 items generating a score of 5 or greater among at least 75% of participants (9/12). The highest and second highest scored items were turnaround times (item 7, mean score 7.1) and readability of the text (item 5, mean score 6.8). Terminology and abbreviations used in the interface (item 10) was the third highest scored item, with a mean score of 6.75. Other items with average scores above 6.0 were the interface’s use of color (item 6, mean score 6.7), easily understood symbols and icons (item 11, mean score 6.6), appropriate number of elements for control (item 2, mean score 6.3), simple visualization (item 15, mean score 6.2), corresponds to expectations (item 8, mean score 6.1), and navigation (item 13, mean score 6.1).

Aspects of the interface that were scored between 5 and 6 were related to its design, such as straightforwardness of visualizations (item 1, mean score 5.8) and consistency of design (item 4, mean score 5.8). In addition, items related to the levels of information provided by the interface were scored similarly (ie, item 3 and item 9) along with that of customization (item 17).

**Figure 2:**
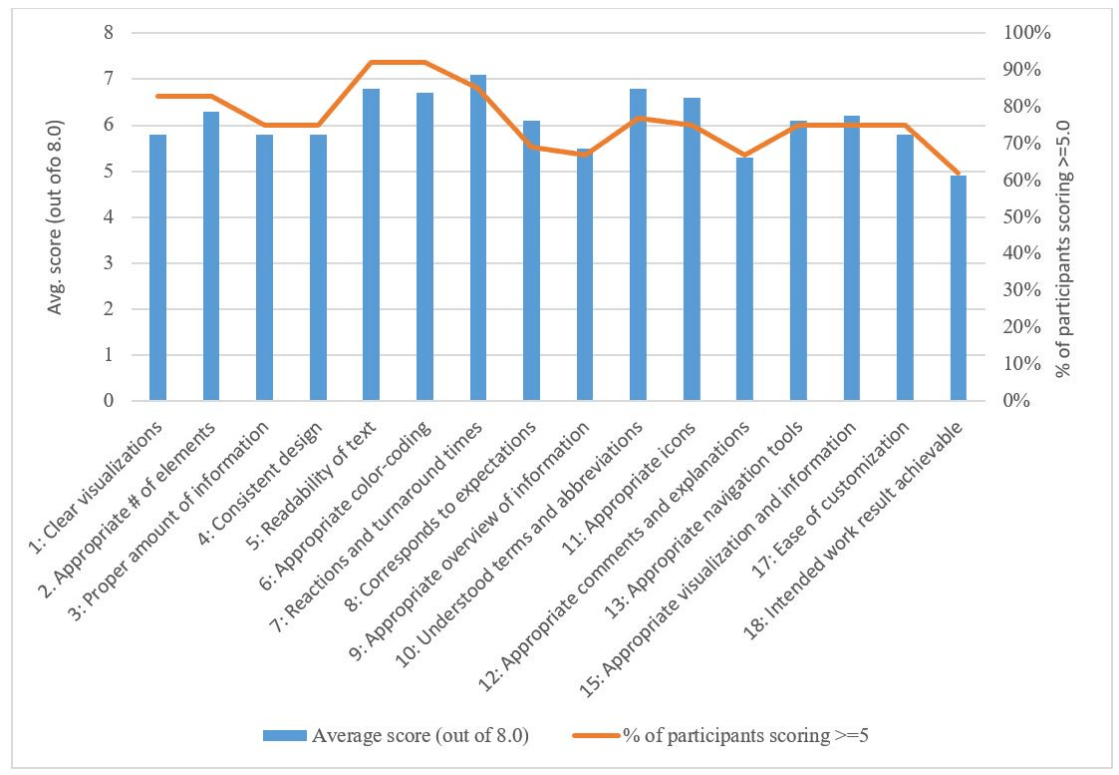
Results by items in the usability questionnaire.

The lowest performing items pertained to the interface’s output. Item 18 (effect of incorrect inputs on intended work results) and item 12 (comments and explanations) scored an average of 4.9 and 5.3, respectively. It is noteworthy that every item, including the aforementioned ones with the lowest scores, scored in the “positive” range. The results for all the items on the questionnaire are shown in Table 2 and Figure 2. The sums of scores from each participant were also calculated. The average score was 109; the median score was 111.5; and scores ranged from 57-142.

**Table 2:** Results by items in the usability questionnaire.

Items		Scores on 8-point Likert scale		Responses from participants (N=12) with scores ≥5 on Likert scale, n (%)
		Mean	Median	
**Suitability for the task**				
	Clear visualizations (item 1)	5.8	6.0	10 (83)
	Appropriate number of elements (item 2)	6.3	7.0	10 (83)
	Proper amount of information (item 3)	5.8	6.0	9 (75)
**Conformity with user expectations**				
	Consistent design (item 4)	5.8	7.0	9 (75)
	Readability of text (item 5)	6.8	7.0	11 (92)
	Appropriate color-coding (item 6)	6.7	7.0	11 (92)
	Reactions and turnaround times (item 7)	7.1	8.0	11 (92)
	Corresponds to expectations (item 8)	6.1	7.0	9 (75)
**Self-descriptiveness**				
	Appropriate overview of information (item 9)	5.5	6.0	8 (67)
	Understood terms and abbreviations (item 10)	6.8	7.5	10 (83)
	Appropriate icons (item 11)	6.6	8.0	9 (75)
	Appropriate comments and explanations (item 12)	5.3	5.0	8 (67)
**Controllability**				
	Appropriate navigation tools (item 13)	6.1	7.0	9 (75)
	Undo single steps (item 14)	N/A^a^	N/A	N/A
	Appropriate visualization of information (item 15)	6.0	6.0	9 (75)
**Suitability for individualization**				
	Undo single steps (item 16)	N/A	N/A	N/A
	Ease of customization (item 17)	5.8	7.0	9 (75)
**Error tolerance**				
	Intended work result achievable (item 18)	4.9	5.0	8 (62)

^a^N/A: not applicable.

## Discussion

### Principal Results

This study tested the usability of a graphical interface in displaying health data from patients’ smartwatches for integration with EHRs; we found that 14 of the 16 categories received above neutral/average scores from the majority of participants. Health care professionals were particularly satisfied with readability of the text and the interface’s speedy response times. Improvements to the interface should prioritize allowing participants more control over data for better customization as per specific user needs. Results from this usability study support the findings from our qualitative interviews [3] as well as other studies in which health care professionals trusted health data from smartwatches and believed those would be helpful in clinical decision making [19]. Previous studies found that health care providers believed that wearable devices could improve health [20] and recommended health data from smartwatches to be incorporated into the convenient and secure environment of EHR systems [3]. Our qualitative study [3] also found that each medical specialty required different types of data and applied those data to different uses. This usability test demonstrated that the interface can satisfy a wide range of user needs. In regard to data visualization, the colors and charts recommended by health care professionals were chosen from differing layouts. The line graph depiction was proven to be the most effective, as it allowed participants to track longitudinal data easily.

### Recommendations for Interface Integration

Although we received positive responses on the interface from participants in our sample, further testing is required to simulate the environment of health care professionals’ typical workflow. We achieved an average aggregate score of 109 from the questionnaire (omitting 2 items). This score is higher than the one reported by another study evaluating a web-based platform (105.8) [18]. Considering these results, an iterative approach will be taken in which the interface will be improved incrementally until a satisfactory threshold for each item is achieved, and aggregate scores improve [21]. Once the interface is finalized, pilot tests will be conducted in clinical settings to ensure that health data from smartwatches are effectively integrated with EHRs, enhancing the way health care professionals utilize data. These pilot tests will determine the true utility of the interface and integrated data. This adoption process is similar to that of EHRs when they were introduced. Although cognitive task analysis was used to reveal how physicians used electronic medical records [14], successful integration of health information technology into the clinical workflow was only achieved when the benefits and barriers of implementation were considered [22]. The EHR system has become an essential vehicle for advancing quality of care [23]. Therefore, it is imperative to ensure that incorporating health data from smartwatches does not disrupt how EHRs are currently utilized but instead modernizes the technology by using the additional data to support clinical decisions and improve care.

### Limitations

Our study had a small sample size and included health care professionals who volunteered to participate. Therefore, results cannot be generalized and may not reflect the opinions of other health care professionals. In addition, participants may have been primed by their exposure to preliminary versions of data charts in the prior qualitative study. Seeing visual elements for a second time that were included in the graphical interface may have positively influenced their perceptions. Although we used mock data, the evaluation was conducted in a test environment; therefore, results may differ if the interface was used during regular clinical workflows. Similarly, in clinical settings, providers may consider the issue of liability in which they may be assumed to be knowledgeable and responsible for the data, which may alter their evaluation of the graphical interface.

### Conclusions

Incorporating health data from smartwatches into EHRs may benefit patient care, but it is important to consider the way in which data are presented to and visualized by health care professionals. Partnering with key stakeholders (health care professionals), who will be the main users of the interface is essential to developing a practical and valuable platform.

## Appendix

Multimedia Appendix 1 Usability study questionnaire along with the omitted questions.

## Abbreviations

EHR: electronic health record
ISO: International Organization for Standardization
