# Supplementary material for: A Simulated Graphical Interface for Integrating Patient-Generated Health Data From Smartwatches With Electronic Health Records: Usability Study
Source: JMIR Hum Factors. 2020 Oct 30;7(4):e19769. doi: 10.2196/19769 (PMC7665942; doi:10.2196/19769)
Supplement: Multimedia Appendix 1 [file humanfactors_v7i4e19769_app1.docx]

Supplement 1. Usability study questions*.

Suitability for the Task

1. The visualizations are

| 1 | 2 | 3 | 4 | 5 | 6 | 7 | 8 |
| --- | --- | --- | --- | --- | --- | --- | --- |
| complicated and  confusing | | | | straightforward and  clearly structured | | | |

1. The interface includes

| 1 | 2 | 3 | 4 | 5 | 6 | 7 | 8 |
| --- | --- | --- | --- | --- | --- | --- | --- |
| unnecessary elements  for control and interaction | | | | appropriate number of elements  for control and interaction | | | |

1. The interface offers

| 1 | 2 | 3 | 4 | 5 | 6 | 7 | 8 |
| --- | --- | --- | --- | --- | --- | --- | --- |
| too much and  unnecessary information | | | | exactly fitting and  necessary information | | | |

Conformity with User Expectations

1. The interface has

| 1 | 2 | 3 | 4 | 5 | 6 | 7 | 8 |
| --- | --- | --- | --- | --- | --- | --- | --- |
| an inconsistent design | | | | a consistent design | | | |

1. The interface contains

| 1 | 2 | 3 | 4 | 5 | 6 | 7 | 8 |
| --- | --- | --- | --- | --- | --- | --- | --- |
| text that is  hard to read | | | | easily readable text | | | |

1. The interface has

| 1 | 2 | 3 | 4 | 5 | 6 | 7 | 8 |
| --- | --- | --- | --- | --- | --- | --- | --- |
| bad color-coding | | | | good color-coding | | | |

1. The interface

| 1 | 2 | 3 | 4 | 5 | 6 | 7 | 8 |
| --- | --- | --- | --- | --- | --- | --- | --- |
| reacts slowly with  unpredictable turnaround times | | | | reacts fast and with  predictable turnaround times | | | |

1. The interface includes interactive elements that

| 1 | 2 | 3 | 4 | 5 | 6 | 7 | 8 |
| --- | --- | --- | --- | --- | --- | --- | --- |
| contradict my  expectations and habits | | | | correspond to my  expectations and habits | | | |

Self-descriptiveness

1. The interface offers

| 1 | 2 | 3 | 4 | 5 | 6 | 7 | 8 |
| --- | --- | --- | --- | --- | --- | --- | --- |
| no overview of  provided information | | | | good overview of  provided information | | | |

1. The interface uses

| 1 | 2 | 3 | 4 | 5 | 6 | 7 | 8 |
| --- | --- | --- | --- | --- | --- | --- | --- |
| vague and unclear terms  and abbreviations | | | | terms and abbreviations  that are easily understood | | | |

1. The interface uses

| 1 | 2 | 3 | 4 | 5 | 6 | 7 | 8 |
| --- | --- | --- | --- | --- | --- | --- | --- |
| ambiguous and unclear  symbols and icons | | | | symbols and icons that  can be easily understood | | | |

1. The interface includes

| 1 | 2 | 3 | 4 | 5 | 6 | 7 | 8 |
| --- | --- | --- | --- | --- | --- | --- | --- |
| unnecessary comments  and explanations | | | | helpful comments  and explanations | | | |

Controllability

1. The interface has

| 1 | 2 | 3 | 4 | 5 | 6 | 7 | 8 |
| --- | --- | --- | --- | --- | --- | --- | --- |
| cumbersome  navigation tools | | | | easy navigation tools | | | |

1. The interface allows to undo single steps

| 1 | 2 | 3 | 4 | 5 | 6 | 7 | 8 |
| --- | --- | --- | --- | --- | --- | --- | --- |
| in a complicated way | | | | easily | | | |

15. The interface provides

| 1 | 2 | 3 | 4 | 5 | 6 | 7 | 8 |
| --- | --- | --- | --- | --- | --- | --- | --- |
| complicated and insufficient  filtering and visualization of information | | | | simple and sufficient visualization  and selection of information | | | |

Suitability for Learning

16**.** The interface allows to undo single steps

| 1 | 2 | 3 | 4 | 5 | 6 | 7 | 8 |
| --- | --- | --- | --- | --- | --- | --- | --- |
| in a complicated way | | | | easily | | | |

Suitability for Individualization

17. The interface

| 1 | 2 | 3 | 4 | 5 | 6 | 7 | 8 |
| --- | --- | --- | --- | --- | --- | --- | --- |
| is difficult to customize | | | | allows view results in a  customized way easily | | | |

Error Tolerance

18. In case of an obviously incorrect input, the intended work result

| 1 | 2 | 3 | 4 | 5 | 6 | 7 | 8 |
| --- | --- | --- | --- | --- | --- | --- | --- |
| cannot be achieved | | | | can be achieved | | | |

*Questions #14 and #16 were not relevant to the current study and therefore, not evaluated
